# An update on the diagnosis and treatment of diabetic somatic and autonomic neuropathy

**DOI:** 10.12688/f1000research.17118.1

**Published:** 2019-02-15

**Authors:** Shazli Azmi, Ioannis N. Petropoulos, Maryam Ferdousi, Georgios Ponirakis, Uazman Alam, Rayaz A. Malik

**Affiliations:** 1 Institute of Cardiovascular Sciences, University of Manchester and Central Manchester NHS Foundation Trust, Manchester Academic Health Science Centre, Manchester, UK; 2 Manchester Diabetes Centre, Manchester University Foundation Trust, Manchester, UK; 3 Weill Cornell Medicine-Qatar, Education City, Qatar Foundation, Doha, Qatar; 4 Department of Eye and Vision Science, Institute of Ageing and Chronic Disease, University of Liverpool, Liverpool, UK; 5 Department of Diabetes and Endocrinology, Royal Liverpool and Broadgreen University NHS Hospital Trust, Liverpool, UK

**Keywords:** diabetes mellitus, peripheral neuropathy, autonomic neuropathy

## Abstract

Diabetic peripheral neuropathy (DPN) is the most common chronic complication of diabetes. It poses a significant challenge for clinicians as it is often diagnosed late when patients present with advanced consequences such as foot ulceration. Autonomic neuropathy (AN) is also a frequent and under-diagnosed complication unless it is overtly symptomatic. Both somatic and autonomic neuropathy are associated with increased mortality. Multiple clinical trials have failed because of limited efficacy in advanced disease, inadequate trial duration, lack of effective surrogate end-points and a lack of deterioration in the placebo arm in clinical trials of DPN. Multifactorial risk factor reduction, targeting glycaemia, blood pressure and lipids can reduce the progression of DPN and AN. Treatment of painful DPN reduces painful symptoms by about 50% at best, but there is limited efficacy with any single agent. This reflects the complex aetiology of painful DPN and argues for improved clinical phenotyping with the use of targeted therapy, taking into account co-morbid conditions such as anxiety, depression and sleep disturbance.

## Diagnosing diabetic peripheral neuropathy: too little too late

The early diagnosis and monitoring of diabetic peripheral neuropathy (DPN) are recommended by both the Toronto consensus ^1^ and the more recent American Diabetes Association (ADA) position statement on DPN ^2^. They recommend the presence of at least one symptom or sign of neuropathy and abnormal neurophysiology for the diagnosis of DPN. Symptom questionnaires, composite neurological scores and quantitative sensory testing (QST) may be used to diagnose DPN ( Table 1). The neuropathy disability score ^3^, a composite measure of neurological deficits (based on Achilles tendon reflexes, 120-Hz vibration, temperature and pin-prick sensation); Toronto Clinical Neuropathy Score ^4^; and the Michigan Neuropathy Screening Instrument ^5^ are validated neurological scores of clinical DPN. Although these tests are adequate tools to screen for DPN, they lack the sensitivity to assess change in clinical trials of relatively short duration (12–24 months). Yet they continue to be advocated as measures of efficacy, despite serial failure to show benefits in clinical trials of DPN.

**Table 1.** Common tests for the assessment of neuropathy.

Type of nerve	Investigation	Advantages and disadvantages
Large fibre	Nerve conduction studies	Gold standard Sensitive, specific, and reproducible and easily standardised Must be done by trained professional
Large and small fibres	Neuropathy disability score	Good predictor for risk of ulceration Subjective Does not detect sub-clinical large fibre damage
Small fibre	Quantitative sensory testing	Reproducible and reliable Subjective
	Skin biopsy	Gold standard for small fibre testing Reliable and reproducible Invasive procedure which needs specialised laboratory service
	Corneal confocal microscopy	Rapid, reproducible, non-invasive Detects small fibre damage and tracks worsening and improvement in small phase 2b clinical trials Requires training to perform

QST ^6^ is a painless, non-invasive means to diagnose small and large fibre dysfunction and is based on impaired thermal, pain and vibration perception, respectively ( Table 1). Elevated vibration perception threshold is a risk factor for foot ulceration and lower-extremity amputation ^7^ but is a subjective test ^8^. Light touch can be assessed by using the 10-g monofilament and is commonly advocated as a screening tool for DPN, although it can only detect advanced neuropathy ^9^ and those at increased risk of amputation ^10^. Diagnosing established DPN is akin to ‘closing the stable door after the horse has bolted’.

Nerve conduction studies assess large fibre function and are currently advocated as the gold standard for a definite diagnosis of DPN ^11^. The typical electrophysiological findings in DPN are reduced amplitude of the compound muscle action potential, slower nerve conduction velocity, prolonged F-wave latency and an altered H-reflex. They are particularly useful for differentiating from other or concomitant neuropathies such as chronic inflammatory demyelinating polyneuropathy (CIDP) ^12^. They are also advocated as a primary end-point to measure therapeutic effect but have equally failed in the majority of clinical trials of DPN ^13^.

Thus, several caveats ought to be carefully considered when these tests are employed to assess change over time and the response to therapies. Although composite scores and QST are resourceful methods to assess neuropathy, they have poor sensitivity and reproducibility ^14^ and low histopathological specificity ^15^. They may be of value in large longitudinal cohort studies as opposed to individual patients or relatively small phase III clinical trials of short duration ^14^. Nerve conduction studies cannot assess small fibre neuropathy and have poor inter-rater reproducibility ^16^, making multi-centre trials difficult ^17^. Indeed, these measures have consistently failed to show meaningful improvements in clinical trials of neuropathy ^18– 20^.

Intra-epidermal nerve density (IENFD) evaluation in skin biopsy offers an objective and more reproducible means to assess small nerve fibre pathology ^21^. IENFD is reduced in pre-diabetes ^22^, predicts incident neuropathy ^23^ and improves with lifestyle intervention ^24^. Unlike neurophysiology, skin biopsy is not confounded by height and weight, although a gender- and age-dependent decline has been reported ^21^. There are published guidelines on the use of skin biopsy to diagnose DPN ^25^. However, wider adoption of skin biopsy is limited by cost, the need for a dedicated processing and assessment facility, and the risk of bleeding and infection following the procedure. Corneal confocal microscopy (CCM) is a powerful, non-invasive ophthalmic imaging end-point for DPN and other neuropathies ^26^. CCM can be used to quantify small fibre pathology with high reproducibility ^27^. Corneal c-fibres form the sub-basal nerve plexus and are highly metabolically demanding ^28^ and hence vulnerable even to transient and minor metabolic perturbation ^22^. Recent studies have established CCM values for the diagnosis ^29– 31^ and prediction ^32, 33^ of DPN on the basis of corneal nerve fibre density and length ^34– 37^. CCM has also shown corneal nerve regeneration in clinical trials before change in other US Food and Drug Administration (FDA)-accepted end-points (for example, neurophysiology and skin biopsy), a finding which has important implications for clinical trial design ^13^.

## Disease modification for diabetic neuropathy

In the 2017 ADA position statement on diabetic neuropathy, early recognition of DPN even in pre-diabetes is recommended ^2, 38, 39^. There are no FDA-approved disease-modifying treatments for the management of DPN, and improved glycaemic control to prevent progression forms the mainstay of treatment. In type 1 diabetes mellitus (T1DM), improved glycaemic control can prevent the development and delay the progression of neuropathy ^40^. However, in T2DM, there is limited evidence that improved glycaemic control can slow down the progression of neuropathy ^41– 43^. The EURODIAB IDDM (European Diabetes Centers Study of Complications in Patients with Insulin-dependent Diabetes Mellitus), which evaluated over 3000 patients with T1DM for a duration of 7 years, identified poor glycaemic control, elevated low-density lipoprotein (LDL) cholesterol and triglycerides, hypertension, obesity and smoking as risk factors for incident DPN ^44^.

In patients with T1DM, treatment with an angiotensin-converting enzyme (ACE) inhibitor ^45^ or in combination with a calcium channel blocker ^46^ has been shown to improve DPN in randomised placebo-controlled trials ^47, 48^. Statins and fibrates have also been shown to prevent the development of DPN ^49, 50^ and have been associated with reduced diabetic foot infection ^51^ and lower-extremity amputation ^52, 53^ and increased rate of foot ulcer healing ^54^. Recent experimental studies advocate the use of combination therapies targeting several pathogenetic pathways as the most effective approach to the treatment of DPN ^55– 57^.

## Painful diabetic peripheral neuropathy

### Diagnosis of painful diabetic peripheral neuropathy

**Figure 1.**
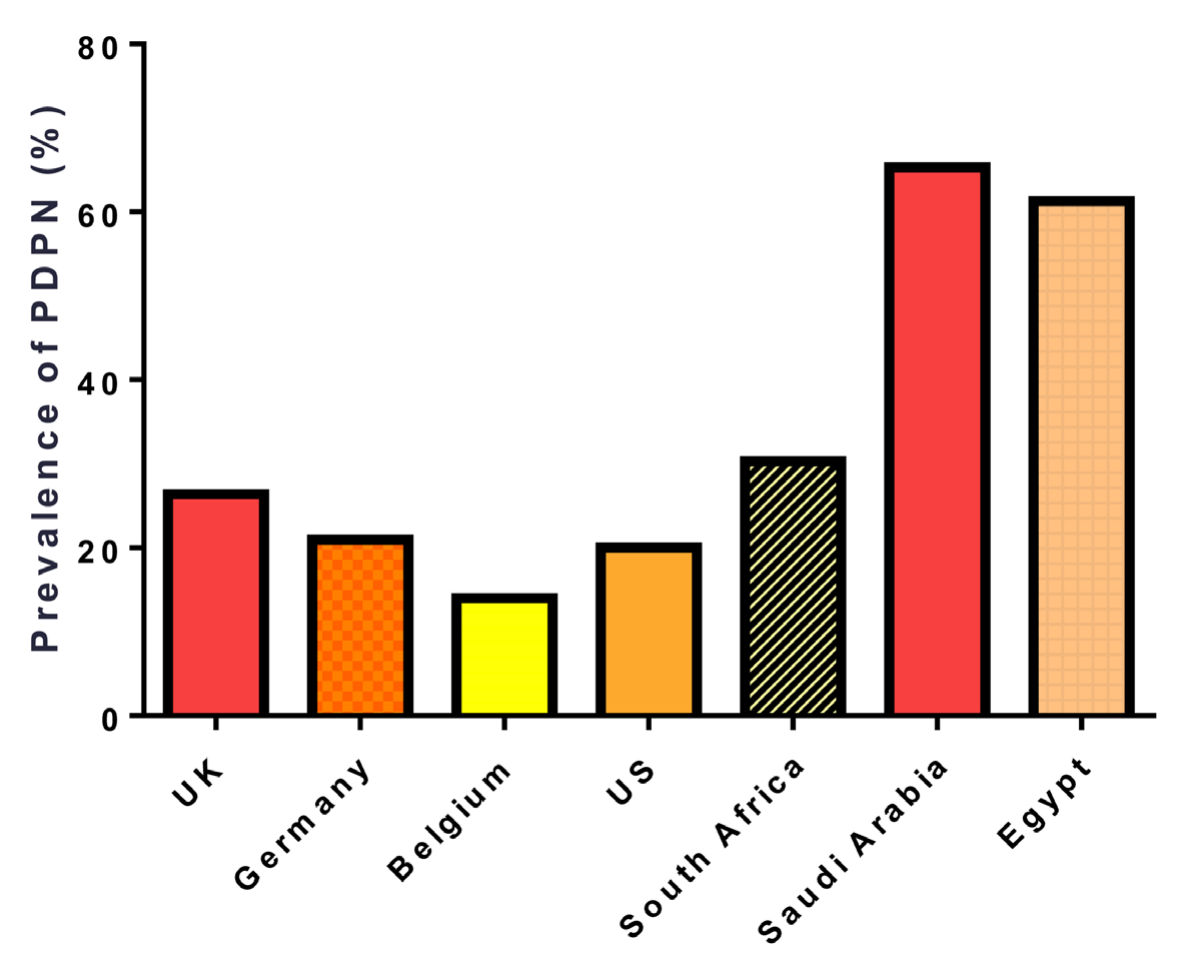
Prevalence of painful diabetic peripheral neuropathy (PDPN) reported from different countries ranges from 14.0 to 65.3% ^61, 64– 69^.

Painful diabetic peripheral neuropathy (PDPN) is a manifestation of small fibre damage ^58– 60^, characterised by burning pain and tingling with nocturnal exacerbation. It has a significant impact on the patient’s quality of life ^61– 63^ and can result in depression, anxiety and sleep disturbance ^62^. Estimates of the prevalence of PDPN range from 14.0 to 65.3% ^61, 64– 69^ ( Figure 1). This difference can be attributed to different populations studied and different diagnostic methods. The prevalence of PDPN is higher in secondary compared with primary care ^70^ and in patients with T2DM compared with T1DM ^61, 64, 71^. It is important to note that for a large proportion (12.5–61.5%) of patients, PDPN remains undiagnosed ^71, 72^. They are often unaware that the pain is related to diabetes and do not report it to their clinician ^72^. Older age, longer duration of diabetes, and the presence of DPN increase the risk for PDPN ^61, 64– 66, 68^; and obesity ^61, 65, 70, 71^, low physical activity ^24, 67^, smoking ^64, 70^, poor glycaemic control ^73, 74^, low high-density lipoprotein (HDL) cholesterol ^61^, and raised LDL cholesterol, triglycerides and creatinine ^67^ are independent risk factors for PDPN. Early diagnosis and intervention may be the key, as a study of subjects with pre-diabetes showed that lifestyle intervention reduced neuropathic symptoms and improved small fibre function and structure ^24^. Therefore, actively screening patients for PDPN, particularly those at high risk, should allow early identification and symptom relief but may also enable timely disease modification.

There are several screening tests that distinguish nociceptive and neuropathic pain. The most common screening tests for PDPN are the Douleur Neuropathique 4 (DN4) questionnaire ^75^, the Leeds Assessment of Neuropathic Symptoms and Signs (LANSS) pain scale ^76^ and the Neuropathic Pain Questionnaire (NPQ) ^77^. The DN4 is composed of 10 questions (seven symptoms and three neurological deficits), and a score of at least 4 has a high sensitivity (83%) and specificity (90%) for PDPN ^78^. The LANSS pain scale is composed of five questions on symptoms and two on neurological deficits, and a score at least 12 has a 85% sensitivity and 80% specificity for PDPN ^76^. The NPQ contains 10 questions on quality of pain and two on change in sensation ^77^ and detects PDPN with 74.7% sensitivity and 77.6% specificity.

## Treatment of painful diabetic peripheral neuropathy

There is no evidence that improvement in glycaemic control improves PDPN; indeed, where rapid and large reductions in HbA1c may precipitate an acute painful neuropathy, the opposite is true ^79^. The traditional approach to managing PDPN is to try different therapies until one works (with minimal side effects). However, improved clinical phenotyping and targeting of therapies based on underlying mechanism(s) may result in better outcomes ^80^. Detailed phenotyping using QST suggests that in patients with an irritable nociceptor compared with a non-irritable nociceptor phenotype, there is a better response to oxcarbazepine, with a number needed to treat (NNT) of 3.9 compared with 6.9 in those with the non-irritable nociceptor ^81^. Identifying abnormalities in rate-dependent depression (RDD), a marker of altered descending inhibitory modulation of pain, may also help to identify patients who will respond optimally to selective norepinephrine reuptake inhibitors for example, duloxetine ^82^.

Tricyclic anti-depressants (TCAs) modulate pain and have analgesic efficacy by indirectly modifying the opioid system in the brain and via serotonergic and noradrenaline neuromodulation, amongst other mechanisms ^83– 85^. Amitriptyline is the most commonly used TCA in PDPN, despite not having a label for its treatment. In a systematic review, Moore *et al*. ^86^ evaluated 17 studies with 1342 participants and concluded that study quality was modest, and most studies had a high risk of bias due to the small participant numbers ^86^. Two serotonin and norepinephrine reuptake inhibitors (SNRIs) are recommended for PDPN: duloxetine and venlafaxine. They exert their effect via inhibiting serotonin and noradrenaline reuptake with potentiation of descending inhibitory pathways ^87^. A Cochrane review including eight randomised controlled trials (n = 2728) showed that duloxetine 60 mg daily was superior to placebo, and the NNT was 5 ^88^. Pregabalin also has FDA approval for PDPN on the basis of a number of randomised controlled trials (RCTs) ^89– 91^. Snedecor *et al*. undertook a comparative meta-analysis of a number of agents to treat PDPN and found pregabalin to be the most efficacious in reducing Visual Analogue Scale (VAS) pain scores ^92^. Duloxetine and pregabalin are both considered first-line therapy by National Institute for Clinical Excellence (NICE) and the 2017 ADA position statement ^2, 93^. Mirogabalin has also recently shown efficacy and good tolerability in a phase II and two phase III clinical trials in PDPN ^94– 96^. Tramadol has an opioid plus SNRI effect. A Cochrane Collaboration review found that the efficacy of tramadol in neuropathic pain was determined in small, inadequately sized studies with a risk of bias ^97^, although a meta-analysis showed an NNT of 4.4. Tapentadol extended-release has also shown efficacy in a number of randomised clinical trials and is recommended by the FDA in PDPN ^98– 101^.

The COMBO-DN (Combination versus Monotherapy of pregabalin and duloxetine in Diabetic Neuropathy) study compared monotherapy with a combination of duloxetine and pregabalin ^102^. The pain outcomes between combination (duloxetine 60 mg daily plus pregabalin 300 mg daily) and high-dose monotherapy (duloxetine 120 mg daily or pregabalin 600 mg daily) were comparable ^102^. In an exploratory post-hoc analysis, high-dose monotherapy was more favourable in patients with severe pain, whereas combination therapy was more beneficial in patients with mild to moderate pain ^103^. In a double-blind RCT with a parallel-group design, analgesic efficacy was found to be comparable between amitriptyline, duloxetine and pregabalin ^104^.

In patients presenting with PDPN, the key is to provide symptom relief, but we would argue that this also represents a window of opportunity for risk factor reduction to limit DPN progression. For symptom relief, pregabalin, gabapentin, duloxetine or amitriptyline can be used first line, and if they are not working or have limited effectiveness because of side effects, then a second-line agent or tramadol can be added in combination ( Table 2). Topical therapies such as a glyceryl trinitrate (GTN) spray ^105^ or patch ^106^ applied to the feet can be considered and give a favourable NNT of about 4 ^107, 108^. There is an evolving argument that the future management of PDPN will follow a more personalised approach using markers such as RDD ^109^, CCM ^110^ and genomics ^111– 113^ to identify specific mechanisms in patients who will respond better to targeted therapies.

**Table 2.** Commonly used therapy for painful diabetic peripheral neuropathy.

Drug class	Agent	Initial dose	Maintenance dose	Comments and common adverse reactions
Anticonvulsants	Pregabalin ^89– 92^	25–75 mg three times a day	300–600 mg daily	Adverse events (AEs): dizziness, somnolence, headache and weight gain Approved for the treatment of painful diabetic peripheral neuropathy (DPN) Psychological dependence
	Gabapentin ^128, 129^	100–300 mg three times a day	900–3600 mg daily	AEs: dizziness, somnolence, ataxia and fatigue Reduce dose if estimated glomerular filtration rate is less than 60 mL/min.
Antidepressants	Duloxetine ^88, 130– 132^	20–30 mg once daily	60–120 mg once daily	Approved for the treatment of painful DPN AEs: somnolence, dizziness, headache, nausea, dry mouth and reduced appetite Avoid in hepatic impairment; avoid with creatinine clearance of less than 30 mL/min.
	Venlafaxine ^133, 134^	37.5 mg once daily	75–225 mg once daily	AEs: nausea, dizziness, constipation, dry mouth, weight loss and constipation
	Amitriptyline ^83, 86^	10–25 mg once daily	25–100 mg once daily	AEs: abdominal pain, headaches, dizziness, insomnia, orthostatic hypotension, anorexia, nausea, urinary retention, constipation, blurred vision, mydriasis, weight gain, xerostomia and somnolence Avoid use in patients older than 60 years of age.
Opioid-like agonists	Tramadol ^97^	50 mg four times a day	200–400 mg four times a day	AEs: constipation, somnolence, nausea, headache and dizziness
	Tapentadol ^98– 101^	50–100 mg four to six times per day; can take 700 mg on first day	600 mg daily	AEs: nausea, dizziness, somnolence, constipation, vomiting and headache Potential for addiction, abuse and misuse
Topical therapies	Capsaicin 0.0075% cream ^135– 138^		Applied three or four times per day	Can be used as an adjunct to oral therapies Use is limited by the frequency of application. Cause denervation, hence may increase risk of foot ulceration
	Lidocaine 5% plaster ^92, 139^		5% for up to 18 hours per day	AEs: application site reactions; otherwise, has fewer side effects than systemic agents Can be used as an adjunct to oral therapies
	Isosorbide dinitrate ^106^		Patch (5 mg) applied at bedtime to the bottom of the feet	AEs: headache. The dose can be halved if this occurs.

## Cardiac autonomic neuropathy

The Toronto consensus panel defined cardiac autonomic neuropathy (CAN) as the impairment of cardiovascular autonomic control in patients with diabetes mellitus after the exclusion of other causes ^114^. CAN occurs in subjects with impaired glucose tolerance (IGT), and abnormal cardiovascular autonomic reflex tests (CARTs) have been reported in up to 7% of patients at diagnosis of T1DM and T2DM ^115– 117^ with the prevalence increasing to 30% (Diabetes Control and Complications Trial/Epidemiology of Diabetes Interventions and Complications, or DCCT/EDIC) after 14 years of T1DM ^118, 119^ and in other studies to 70% after 15 years ^120^. CAN is an independent risk factor for mortality ^114^ and was the strongest risk factor for all-cause mortality in the EURODIAB study (T1DM) and an independent risk factor for mortality in the Action to Control Cardiovascular Risk in Diabetes (ACCORD) study (T2DM) ^121, 122^. A meta-analysis of 15 longitudinal studies reported an association between CAN and increased mortality ^123^. A meta-analysis of 12 studies identified silent myocardial ischaemia (SMI) in 20% of patients with CAN compared with 10% in those without CAN ^117^. CAN is also associated with left ventricular dysfunction ^124, 125^ and independently predicts the progression of diabetic nephropathy ^126^.

## Diagnosis of cardiac autonomic neuropathy

Screening for CAN is recommended at diagnosis in patients with T2DM and after 5 years for those with T1DM. Signs and symptoms of CAN should be assessed in patients with microvascular complications and in patients with hypoglycaemia unawareness ^2, 127^. The diagnosis of CAN includes the documentation of the symptoms and signs ( Table 3), but there is a weak correlation between symptoms and autonomic deficits ^120, 140^ and symptoms may be subtle like intermittent palpitations and exercise intolerance. CAN may initially be asymptomatic with the only sign being decreased heart rate variability with deep breathing, which can progress to a resting tachycardia (>100 bpm). In patients who are symptomatic (resting tachycardia with a history of poor glucose control) or where the diagnosis of CAN is very likely, the ADA position statement advises no additional testing ^2^. It is important to exclude other causes such as idiopathic orthostatic hypotension (OH), syncope and postural orthostatic tachycardia syndrome (POTS) ^141^. CARTs includes heart rate response to deep breathing, standing and the Valsalva manoeuvre.

**Table 3.** Symptoms and signs of diabetic autonomic neuropathy.

**Cardiac autonomic neuropathy** • Resting tachycardia or fixed heart rate or both • Loss of circadian rhythm of blood pressure • Increase in nocturnal systolic blood pressure compared with daytime • Orthostatic hypotension • Exercise intolerance • Syncope and light headedness • Intra-operative cardiovascular lability • ‘Silent ischemia’ and ‘painless’ myocardial infarction • Arrhythmias • **Urogenital autonomic neuropathy** Bladder dysfunction • Increase in frequency and urgency particularly during the night • Urinary hesitancy and weak stream • Dribbling of urine and involuntary urination • Urinary incontinence Sexual dysfunction • Male: erectile dysfunction, decreased libido and abnormal ejaculation • Female: decreased sexual desire and arousal, increased pain during intercourse and inadequate lubrication **Gastrointestinal autonomic neuropathy** • Nausea/vomiting • Bloating • Inability to eat a full meal • Profuse and watery diarrhoea (nocturnal) • Constipation

## Management of autonomic neuropathy

Poor glycaemic control and longer diabetes duration are established risk factors for CAN ^42, 118, 119^. The DCCT showed that intensive glycaemic control in patients with T1DM reduced the development of CAN by 45% ^118^. Hypertension, obesity, hyperlipidemia and smoking have also been implicated in the development of CAN ^42, 117, 120, 142– 144^, and the Steno-2 trial showed that intensified multifactorial treatment in patients with T2DM reduced the risk of CAN progression by 68% ^145, 146^. There are no FDA-approved disease-modifying treatments to reverse CAN. A small early study found favourable effects of alpha-lipoic acid (ALA) on CAN ^147^, however, more recently a study to evaluate triple anti-oxidant therapy (allopurinol (300 mg once daily), ALA (600 mg twice daily) and nicotinamide (750 mg twice daily)) in patients with mild to moderate CAN found no benefit ^148^.

## Orthostatic hypotension

Symptoms of OH occur on standing and include light-headedness, weakness, faintness and syncope. OH is defined by a blood pressure decrease on standing of greater than 20/10 mm Hg (a decrease of greater than 30/15 for those with blood pressure of greater than 150/90) without an appropriate increase in heart rate (<15 bpm) ^149^. Treatment of OH involves a review of medication, fluid and salt repletion and encouragement of physical activity and exercise to avoid deconditioning ^150, 151^. Fludrocortisone is not FDA-approved for OH. It works through sodium retention and constriction of partially denervated blood vessels, but there are concerns over supine hypertension, hypokalaemia, congestive cardiac failure and peripheral oedema ^152^. Both midodrine and droxidopa are approved by the FDA for the treatment of symptomatic neurogenic OH ^153^.

## Gastroparesis

Gastroparesis is defined as the delayed removal of stomach contents in the absence of a physical obstruction ^154^. Gastric emptying should be assessed with scintigraphy 4 hours after food intake of digestible solids at 15-min intervals. Dietary modification with frequent small meals and prokinetics are recommended to increase gastric motility. Metoclopramide is the only FDA-approved drug for the treatment of gastroparesis. However, limited efficacy and the risk of tardive dyskinesia have led the FDA and European Medicines Agency to advise use for a maximum of 5 days. New therapies are being investigated and include motilin receptor agonists, ghrelin receptor agonists, and neurokinin receptor antagonists. Mechanical options for intervention include transpyloric stenting, gastric electrical stimulation, and gastric per-oral endoscopic myotomy; in severe intractable gastroparesis, laparoscopic pyloroplasty or gastrectomy may be options ^155^.

## Diabetic diarrhoea

Diabetic diarrhoea is a troublesome gastrointestinal complication which is characterised by watery painless diarrhoea, particularly at night. Other causes of diarrhoea must be excluded, especially therapy with metformin which is often overlooked, and pancreatic exocrine insufficiency (faecal fat of greater than 6 g/72 hours). Pharmacological therapies include antidiarrhoeal agents (for example, Lomotil or Imodium), antibiotics (tetracycline or metronidazole) to eradicate bacterial overgrowth, somatostatin analogues (octreotide), and selective serotonin 5-hydroxy tryptamine type 3 (HT3) receptor antagonists (Ramosetron) ^156, 157^.

## Bladder disturbance

Bladder dysfunction may occur in up to 50% of patients with diabetes due to urogenital autonomic neuropathy ^141^. The earliest manifestation includes increased initiating threshold for the micturition reflex followed by decreased detrusor activity and incomplete bladder emptying. Treatment includes suprapubic pressure, antimuscarinic medication (oxybutynin 5–30 mg 3 times a day; tolterodine 2–8 mg twice a day) for detrusor hyperreflexia and parasympathomimetic medication to reduce detrusor contractility, and intermittent self-catheterisation ^2^. It is important to note that none of the medications are FDA-approved for neurogenic bladder and they have approval only for non-neurogenic overactive bladder or other lower urinary tract symptoms.

## Erectile dysfunction

Erectile dysfunction (ED) is a common manifestation in men with diabetes ^141^. It may be three times more prevalent, occur 10 to 15 years earlier and is more severe and less responsive to treatment compared with those without diabetes ^158^. ED is associated with a higher HbA1c, presence of metabolic syndrome, hypertension, atherogenic dyslipidemia, lower estimated glomerular filtration rate, higher albumin/creatinine ratio and more severe small fibre neuropathy in men with T1DM ^159– 161^. Sexual dysfunction is also more common in women with diabetes, and 47% of women with diabetic neuropathy had sexual dysfunction ^162^. Reduced sexual arousal, decreased lubrication and painful intercourse are the most common symptoms of sexual dysfunction in females with diabetes. Recent recommendations include active smoking cessation (improves ED by about 30%), treatment of those with testosterone deficiency and treatment with statins. 5-phosphodiesterase inhibitors, intra-cavernosal and transurethral prostaglandins, and penile implants can be used for more severe cases ^163– 165^. There is about a 50% non-responder rate in patients with diabetes as they are less likely to respond to PDE5 inhibitors ^166^. Additional modalities such as low-intensity extracorporeal shock wave therapy show promise ^167– 169^ but require larger and better designed clinical trials. There are several explanations for the lack of response ^166^, but it may also reflect a more severe neurogenic component to ED in these patients ^159^. Indeed, in a recent study, the assessment of nocturnal penile tumescence and rigidity, which reflects predominantly neurogenic abnormalities, had an area under the curve (AUC) of 0.860 in differentiating sildenafil responders from non-responders ^170^.
